# *Candida auris* vs. Non-*Candida auris* Candidemia in Critically Ill Patients: Clinical Outcomes, Risk Factors, and Mortality

**DOI:** 10.3390/jof11080552

**Published:** 2025-07-24

**Authors:** Ezgi Gülten, Güle Çınar, Elif Mukime Sarıcaoğlu, İrem Akdemir, Afife Zeynep Yılmaz, Elif Hilal Saldere, Fügen Yörük

**Affiliations:** Department of Infectious Diseases and Clinical Microbiology, Faculty of Medicine, Ankara University, 06230 Ankara, Turkey; ezgioztop@gmail.com (E.G.); gbinjune@gmail.com (G.Ç.); elifmozturk@gmail.com (E.M.S.); afifezy@hotmail.com (A.Z.Y.); saldereelif@gmail.com (E.H.S.); fugenyoruk@gmail.com (F.Y.)

**Keywords:** antifungal resistance, *Candida auris*, candidemia, intensive care unit, mortality predictors

## Abstract

**Background:***Candida auris* (now *Candidozyma auris*) is an emerging pathogen that causes nosocomial candidemia, particularly in intensive care unit (ICU) settings. Its high resistance rates, prolonged environmental persistence, and outbreak potential underscore the need for robust comparative studies with non-*auris Candida* species (NACS). **Methods:** In this retrospective, case–control study, adult ICU patients with candidemia were enrolled between April 2022 and October 2024. Clinical data, risk factors, and mortality at 14, 30, and 90 days were compared between the *C. auris* and NACS groups. Univariate and multivariate logistic regression analyses were performed to identify mortality-associated factors. **Results:** Of the 182 patients analyzed, candidemia due to *C. auris* was identified in 33 (18.1%) cases, while 149 (81.9%) cases involved NACS. Fluconazole resistance (*p* < 0.001), prior antifungal exposure (*p* = 0.003), urinary catheter use (*p* = 0.040), and the length of ICU stay before the onset of candidemia (*p* < 0.001) were significantly higher in the *C. auris* cases. However, mortality rates at 14, 30, and 90 days were similar between the groups (*p* = 0.331, 0.108, and 0.273, respectively). The Sequential Organ Failure Assessment score was the only consistent independent predictor of mortality at all time points. In the NACS cases, the Pitt Bacteremia Score and sepsis also predicted 30- and 90-day mortality. While late recurrence was more frequent in the cases of *C. auris*, early recurrence and other risk factors were similar between the groups. **Conclusions:** *C. auris* candidemia was associated with higher fluconazole resistance, prior antifungal use, longer ICU stay, more frequent urinary catheterization, and later recurrence than the NACS cases. However, the mortality rates at 14, 30, and 90 days were comparable. Outcomes were primarily influenced by illness severity rather than the infecting *Candida* species, highlighting the importance of timely therapy, stewardship, and infection control.

## 1. Introduction

Candidemia represents a significant proportion of nosocomial bloodstream infections (BSIs), particularly in critically ill patients admitted to intensive care unit (ICU) settings, with attributable mortality rates up to 40.4% despite advances in antifungal therapy [1]. Due to the increasing prevalence of antifungal resistance and the rising incidence of non-*albicans Candida* species, the epidemiology of candidemia has undergone significant changes in recent years [1]. Among these emerging species, *Candida auris*, recently reclassified as *Candidozyma auris*, has gained global attention due to its unique characteristics, including its multidrug resistance profile, high transmissibility, and ability to cause healthcare-associated outbreaks [2].

*Candida auris* was first identified in 2009 and has since been reported in multiple countries across different continents [3]. Whole-genome sequencing analyses have demonstrated that distinct clonal lineages of *C. auris* have independently emerged and spread locally in Asia, South Africa, and South America [4]. These findings underscore the dynamic nature of *C. auris* epidemiology and its capacity for rapid regional dissemination. Unlike other *Candida* species, *C. auris* demonstrates an exceptional ability to persist in hospital environments, colonize patients for prolonged periods, and resist conventional disinfection procedures. More concerningly, *C. auris* exhibits high resistance rates to fluconazole, variable susceptibility to echinocandins, and, in some cases, reduced susceptibility to amphotericin B [5,6].

Despite increasing awareness, comparative data focusing on ICU populations remain limited. ICU patients represent a high-risk group due to prolonged hospitalization, a higher use rate of indwelling devices, and prior antibiotic or antifungal exposure—all recognized risk factors for *C. auris* acquisition [6,7,8]. Moreover, a recent surveillance study suggested that the burden of *C. auris* is underrecognized due to misidentification and a low index of suspicion in non-endemic regions [7]. Data comparing the clinical outcomes and mortality associated with *C. auris* candidemia versus candidemia caused by non-*auris Candida* species (NACS) remain limited and somewhat conflicting. Some studies have suggested that *C. auris* candidemia is associated with worse clinical outcomes, prolonged hospitalization, and higher mortality, while others indicate comparable outcomes to those seen in patients with candidemia due to NACS [9,10]. Given these discrepancies, further research is needed to elucidate whether *C. auris* represents an independent risk factor for poor clinical outcomes or whether its impact is primarily driven by host-related factors, delayed diagnosis, or suboptimal antifungal therapy.

In light of the aforementioned gaps, we aimed to compare the clinical characteristics, risk factors, and outcomes of *C. auris* candidemia versus NACS candidemia in ICU patients. Our findings reveal that *C. auris* candidemia was characterized by greater antifungal resistance, more frequent prior antifungal use, longer ICU stay, more common urinary catheterization, and late recurrence. Notably, the mortality rates at 14, 30, and 90 days did not differ significantly, and illness severity, as reflected by Sequential Organ Failure Assessment (SOFA) scores, remained the strongest predictor of mortality regardless of the infecting species. These findings highlight the need for risk-based clinical management and sustained infection prevention efforts to reduce the burden of *C. auris* in ICU settings.

## 2. Materials and Methods

### 2.1. Study Design and Setting

Ankara University Hospitals provide tertiary healthcare services with a capacity of 1528 inpatient beds and 192 ICU beds across two campuses. In our hospitals, the first patient colonized with *C. auris* was identified on 7 April 2022, and since then, active surveillance for *C. auris* has been implemented. In this case–control study, which aimed to compare the clinical outcomes, risk factors, and mortality in ICU patients with *C. auris* vs. NACS candidemia, cases hospitalized in our hospitals between 7 April 2022 and 1 October 2024 were evaluated.

### 2.2. Patient Selection, Inclusion, and Exclusion Criteria

The study population comprised critically ill adult patients (aged ≥ 18 years) with candidemia. Patients were defined as “critically ill” when admitted to the ICU with at least one acute organ dysfunction requiring life-support interventions, such as mechanical ventilation (MV) for respiratory failure, vasopressor support for hemodynamic instability, or renal replacement therapy for acute kidney injury.

Patients were eligible for inclusion if they were aged 18 years or older, diagnosed with candidemia during their ICU stay with at least one positive blood culture for *Candida* species (processed using the BD BACTEC™ FX (Franklin Lakes, NJ, USA) automated blood culture system), and exhibited clinical signs and laboratory evidence consistent with systemic infection. These included fever (≥38 °C); hypothermia (<36 °C); chills or rigors; new-onset hypotension (systolic blood pressure <90 mmHg or mean arterial pressure <65 mmHg); altered mental status; tachycardia (>90 bpm); tachypnea (>20 breaths/min); leukocytosis (>12,000/mm^3^); leukopenia (<4000/mm^3^); or elevated inflammatory markers, such as C-reactive protein or procalcitonin. Patients were excluded if they had polymicrobial BSIs, defined as concurrent isolation of *Candida* species and one or more bacterial or fungal pathogens from blood cultures within 48 h; lacked clinical or laboratory evidence of systemic infection at the time of yeast detection; were discharged from the ICU before the onset of candidemia; or had insufficient clinical or microbiological documentation.

A total of 201 patients were initially screened. Of these, 19 were excluded due to polymicrobial infections (n = 9), the absence of clinical signs and laboratory evidence of infection (n = 4), ICU discharge prior to a diagnosis of candidemia (n = 4), or insufficient clinical/microbiological data (n = 2). The final cohort included 182 patients.

### 2.3. Definitions, Data Collection, and Outcome Measures

#### 2.3.1. Definitions

Sepsis was defined according to the Sepsis-3 criteria, characterized by life-threatening organ dysfunction caused by a dysregulated host response to infection [11]. Candidemia episodes occurring at least 30 days apart, with documented clinical and microbiological resolution in the interim, were categorized as late recurrence, whereas episodes recurring within 8–29 days were defined as early recurrence [12].

The assessment of mortality was stratified into 14-, 30-, and 90-day intervals to reflect distinct clinical trajectories: 14-day mortality indicates early deaths likely attributable to the acute phase of infection; 30-day mortality reflects short-term outcomes and treatment response; and 90-day mortality encompasses late complications and overall prognosis. This approach is consistent with established practices in candidemia research [13,14,15].

#### 2.3.2. Data Collection

Clinical and laboratory data were retrieved from electronic medical records and patient files. For each patient, their demographic and clinical characteristics, including age, sex, and underlying comorbidities, such as diabetes mellitus (DM), cardiovascular disease (CVD), chronic kidney disease (CKD), solid organ malignancy (SOM), and hematologic malignancy (HM), were recorded. Immunosuppressive status and surgical history were assessed, including abdominal surgery within 30 days prior to candidemia, recent use of corticosteroids or other immunosuppressive therapies within seven days, and history of solid organ transplantation (SOT) or hematopoietic stem cell transplantation (HSCT). Data regarding organ support and ICU-related interventions were collected, including the use of hemodialysis or continuous renal replacement therapy (CRRT), the length of ICU stay before candidemia onset, the presence of central venous catheter (CVC), urinary catheter (UC), and MV, and receipt of parenteral nutrition.

Severity of illness was evaluated using the SOFA score and Pitt Bacteremia Score (PBS), and the presence of sepsis at the time of candidemia diagnosis was noted. Infection-related variables included classification as primary or secondary candidemia, presumed sources of secondary infection, and antifungal resistance profiles. Antibacterial and antifungal exposure within 30 days prior to candidemia and antifungal treatment during the candidemia episode were recorded, along with the time to appropriate antifungal initiation and the time to blood culture negativity. The assessed clinical outcomes included early and late recurrence and all-cause mortality at 14, 30, and 90 days.

#### 2.3.3. Outcome Measures

The primary outcome of this study was 30-day all-cause mortality. Secondary outcomes included 14-day and 90-day mortality, early and late recurrence of candidemia, time to initiation of appropriate antifungal therapy, time to blood culture negativization, and the type and adequacy of antifungal treatment. Additionally, the clinical characteristics and associated risk factors for candidemia caused by *C. auris* versus NACS were analyzed.

### 2.4. Microbiological Identification and Antifungal Susceptibility Testing

#### 2.4.1. Microbiological Identification

Blood cultures were processed using the BD BACTEC™ FX automated blood culture system, following standard aerobic and anaerobic incubation protocols. Direct Gram staining was performed from the positive blood culture broth to confirm the presence of yeast cells. Positive blood culture bottles that flagged for fungal pathogens were subcultured onto Sabouraud dextrose agar ([SDA], containing 40 g/L dextrose, 10 g/L peptone, and 15 g/L agar; final pH 5.6 ± 0.2) and incubated at 35–37 °C for 24–48 h. Following incubation, yeast colonies were assessed based on their macroscopic appearance and microscopic morphology. Species-level identification of *Candida* isolates was performed using matrix-assisted laser desorption/ionization time-of-flight mass spectrometry (MALDI-TOF MS; Bruker Daltonics, Bremen, Germany), based on spectral pattern matching against a validated reference database.

#### 2.4.2. Antifungal Susceptibility Testing

Antifungal susceptibility testing was performed using the broth microdilution method according to the European Committee on Antimicrobial Susceptibility Testing (EUCAST) definitive guideline E.DEF 7.4 [16]. Yeast suspensions were prepared from *Candida* colonies grown on SDA, suspended in sterile 0.9% saline, and adjusted to a final concentration of 0.5 to 2.5 × 10^5^ colony-forming units per milliliter, corresponding to a spectrophotometric absorbance of 0.08 to 0.1 at 530 nm. The antifungal agents tested—fluconazole, caspofungin, micafungin, anidulafungin, and amphotericin B—were dissolved in sterile distilled water or dimethyl sulfoxide, depending on solubility, and stored at 2–8 °C or −20 °C, as recommended. Serial twofold dilutions of each antifungal agent were prepared in 96-well flat-bottom microdilution plates containing Roswell Park Memorial Institute (RPMI) 1640 medium (containing 2.0 g/L glucose, buffered with 0.165 mol/L morpholinepropanesulfonic acid [MOPS], adjusted to a pH of 7.0 ± 0.1, and free of phenol red and sodium bicarbonate). Microdilution plates were inoculated with the standardized yeast suspension to achieve a final volume of 100 µL per well and incubated at 35 °C for 24 h. Each test included growth control wells (medium plus inoculum without antifungal agents) and sterility control wells (medium only). The minimum inhibitory concentration (MIC) was defined as the lowest drug concentration resulting in 50% growth inhibition compared to the growth control for azoles, and complete (100%) inhibition for amphotericin B and echinocandins, as assessed visually. Susceptibility to fluconazole, echinocandins, and amphotericin B was interpreted using species-specific clinical breakpoints defined by the EUCAST guidelines, while for *C. auris* isolates, tentative epidemiological cutoff values and expert consensus thresholds recommended by the Clinical and Laboratory Standards Institute (CLSI) were used due to the lack of universally established breakpoints [17,18]. Accordingly, fluconazole resistance for *C. auris* was defined as MIC ≥ 32 µg/mL, while echinocandin resistance was defined as MIC ≥ 4 µg/mL for anidulafungin and micafungin, and ≥2 µg/mL for caspofungin; amphotericin B resistance was defined as MIC ≥ 2 µg/mL. For NACS, antifungal susceptibility interpretations varied among *Candida* species. For instance, fluconazole resistance was defined as MIC > 4 µg/mL for *Candida albicans* and *Candida parapsilosis*, and >16 µg/mL for *Candida glabrata*. Echinocandin resistance, particularly to micafungin and anidulafungin, was interpreted according to species-specific MIC thresholds: for anidulafungin, resistance was defined as >0.016 µg/mL for *C. albicans*, >0.06 µg/mL for *C. glabrata*, and >4 µg/mL for *C. parapsilosis*; for micafungin, the corresponding thresholds were >0.03 µg/mL for *C. albicans* and *C. glabrata*, and >4 µg/mL for *C. parapsilosis*. Amphotericin B resistance was defined as MIC > 1 µg/mL for all NACS.

### 2.5. Statistical Analysis

Statistical analyses were conducted using IBM SPSS Statistics for Windows, Version 27.0.1.0 (IBM Corp., Armonk, NY, USA). Continuous variables were presented as medians with interquartile ranges (IQRs: 25th–75th percentiles) and compared using the Mann–Whitney U test due to non-normal distribution. Categorical variables were summarized as counts and percentages and analyzed using Pearson’s chi-squared test or Fisher’s exact test where appropriate.

To identify variables associated with 14-, 30-, and 90-day mortality, univariate analyses were initially performed. Variables with a *p*-value < 0.05 in univariate analysis were included in multivariate logistic regression models to determine independent predictors of mortality. A backward stepwise selection method was used to eliminate non-significant variables. The odds ratio (OR) was reported with the 95% confidence interval (CI), and a two-sided *p*-value < 0.05 was considered statistically significant.

## 3. Results

### 3.1. Demographic and Clinical Variables

A total of 182 patients with candidemia were included in this study, of whom 33 (18.1%) had *C. auris* candidemia and 149 (81.9%) had candidemia due to other *Candida* species. Among the NACS candidemia cases, 53 (35.6%), 52 (34.9%), 28 (18.8%), 6 (4%), 5 (3.4%), 3 (2%), and 2 (1.3%) were attributed to *Candida albicans*, *Candida parapsilosis*, *Candida glabrata*, *Candida krusei*, *Candida tropicalis*, *Candida kefyr*, and *Candida dubliniensis*, respectively. The median age of all patients was 69 years (59.8–80), and the median age of patients in the *C. auris* group was significantly lower compared to those with NACS candidemia (65 [50–71.5] years vs. 70 [62–81] years, *p* = 0.003). Among all candidemia cases, 96 (52.7%) were male, and there was no statistically significant difference between the two groups in terms of sex distribution (*p* = 0.166).

The presence of comorbidities and other underlying conditions, including DM, CVD, CKD, SOM, and HM, abdominal surgery within 30 days, corticosteroid and other immunosuppressive treatments within 7 days, the need for hemodialysis and CRRT, and a previous history of SOT or HSCT did not differ significantly between the two groups (*p* = 0.758, *p* = 0.819, *p* = 0.099, *p* = 0.747, *p* = 0.372, *p* = 0.684, *p* = 0.431, *p* = 0.690, *p* = 0.889, *p* = 0.963, and *p* = 0.490, respectively). In addition, based on a detailed review of patient charts, none of the patients received immunosuppressive agents during the candidemia episode, which was defined as the period starting from the onset of candidemia-related clinical signs and symptoms to either clinical resolution or death. For all patients, the median SOFA and PBS values were 6 (4–8) and 4 (3–6), respectively, with no significant difference between the *C. auris* and NACS groups (*p* = 0.693 and *p* = 0.827). However, sepsis was significantly more frequent in patients with *C. auris* candidemia compared to those with candidemia due to NACS (66.7% vs. 45%, *p* = 0.024).

### 3.2. ICU-Related Factors

The duration of hospitalization and the length of ICU stay prior to candidemia were significantly longer in patients with *C. auris* candidemia (49 [35.5–104] vs. 26 [12–41] days, *p* < 0.001, and 35 [17.5–89.5] vs. 13 [3.5–30] days, *p <* 0.001). The proportion of patients with an indwelling UC was significantly higher in the *C. auris* group (93.9% vs. 78.5%, *p* = 0.040), whereas the use of CVC and MV was similar between the two groups (*p* = 0.393 and *p* = 0.960, respectively). Additionally, parenteral nutrition therapy did not differ significantly between the two groups (*p* = 0.301). Prior antibiotic exposure within 30 days did not differ between the groups (*p* = 0.372); however, previous antifungal use during the same period was significantly higher in the *C. auris* group (*p* = 0.003).

### 3.3. Antifungal Resistance, Treatment Outcomes, and Recurrence

Antifungal susceptibility testing revealed that fluconazole resistance was significantly higher in *C. auris* isolates compared to NACS (93.9% vs. 26.8%, *p* < 0.001). However, resistance rates to echinocandins and amphotericin B did not differ significantly between the groups (*p* = 0.450 and *p* = 0.507, respectively). In the *C. auris* group, excluding three patients who died before the initiation of appropriate treatment, 93.3% (n = 28) received echinocandins, and 6.7% (n = 2) received amphotericin B. In the NACS group, after excluding 22 patients who died before antifungal therapy initiation, 84.3% (n = 107) were treated with echinocandins, 11.8% (n = 15) with fluconazole, and 3.9% (n = 5) with amphotericin B. The time from the onset of candidemia to the initiation of appropriate antifungal therapy was comparable between the groups (48 [24–73] h, *p* = 0.925). The median time to negative conversion of blood cultures following antifungal treatment did not differ significantly (5 [4–7] vs. 5 [3–8] days, *p* = 0.720). The rate of early recurrence showed no significant variation between the two groups, whereas late recurrence was more frequently observed in patients with *C. auris* candidemia (*p* = 0.118 and *p* < 0.001).

### 3.4. Mortality

The mortality rates at 14, 30, and 90 days were 44%, 66.5%, and 79.7%, respectively. There were no statistically significant differences in the 14-, 30-, or 90-day mortality rates between the *C. auris* and NACS groups (*p* = 0.331, *p* = 0.108, and *p* = 0.273, respectively). Detailed data regarding the demographic and clinical features of all patients and a comparison of the patients with *C. auris* and NACS candidemia are presented in Table 1.

#### 3.4.1. 14-Day Mortality

At 14 days, 80 (44%) patients did not survive, including 12 (36.4%) *C. auris* and 68 (45.6%) NACS candidemia cases. The univariate analyses indicated that the SOFA score was a significant predictor of 14-day mortality in both *C. auris* and NACS candidemia patients (*p* < 0.001, *p* = 0.011, and *p* < 0.001, respectively). The PBS and sepsis also predicted 14-day mortality in both groups; however, the same association was not observed for *C. auris* candidemia patients (*p* = 0.001 and *p* < 0.001, *p* = 0.726 and *p* = 0.446, *p* < 0.001 and *p* < 0.001, respectively). The prevalence of SOM was found to be significantly higher in the non-survivor *C. auris* candidemia patients (*p* = 0.008). Additionally, among the survivors in the *C. auris* group, the duration of ICU stay before candidemia onset was significantly longer compared to non-survivors (*p* = 0.044) (Table S1).

In the NACS group, independent predictors of 14-day mortality included the SOFA score (OR: 1.254, 95% CI: 1.097–1.433, *p* = 0.001) and the presence of sepsis (OR: 2.339, 95% CI: 1.069–5.118, *p* = 0.033). For the *C. auris* group, the SOFA score was also an independent predictor of 14-day mortality (OR: 1.378, 95% CI: 1.210–1.568, *p* < 0.001).

#### 3.4.2. Thirty-Day Mortality

At 30 days, 121 (66.5%) patients did not survive, including 18 (54.5%) *C. auris* and 103 (89.1%) NACS candidemia cases. In the *C. auris* group, the non-survivors were older than the survivors (*p* = 0.016). The SOFA score and PBS were significantly associated with 30-day mortality in all candidemia patients and the NACS group (*p* < 0.001, and *p* < 0.001, *p* = 0.001, and *p* < 0.001, respectively). However, in the *C. auris* group, the SOFA score and PBS did not demonstrate a predictive effect on 30-day mortality (*p* = 0.062, and *p* = 0.361). In addition, sepsis was a 30-day mortality predictor for only NACS candidemia cases (*p* = 0.043). For all candidemia patients and the NACS group, the rate of CRRT was higher (*p* = 0.010, and *p* = 0.033) (Table 2).

Multivariate analysis for the total cohort identified the SOFA score (OR: 1.138, 95% CI: 1.016–1.273, *p* = 0.025) and PBS (OR: 1.163, 95% CI: 1.015–1.334, *p* = 0.030) as independent predictors of 30-day mortality. In the NACS candidemia cases, PBS remained significant (OR: 1.202, 95% CI: 1.027–1.407, *p* = 0.022). In the *C. auris* group, multivariate analysis revealed that none of the variables were associated with 30-day mortality.

#### 3.4.3. Ninety-Day Mortality

At 90 days, 145 (79.7%) of all patients did not survive, including 24 (72.7%) *C. auris* and 121 (81.2%) NACS candidemia cases. In the entire study cohort, the age of non-survivors was found to be higher than that of survivors (*p* = 0.031). Both the SOFA score and PBS were significant in predicting mortality in all candidemia patients and the NACS group (*p* = 0.002, and *p* < 0.001, *p* = 0.001, and *p* < 0.001, respectively); however, this association was not present in the *C. auris* cases (*p* = 0.890, and *p* = 0.290). Among all candidemia cases, the presence of SOM was higher in the non-survivor subgroup (*p* = 0.028) (Table S2).

According to multivariate logistic regression analysis for the entire cohort, PBS was the only significant predictor of 90-day mortality (OR: 1.258, 95% CI: 1.064–1.488, *p* = 0.007). In the NACS candidemia cases, PBS remained a strong predictor (OR: 1.270, 95% CI: 1.047–1.541, *p* = 0.015). No variable independently predicted 90-day mortality in the *C. auris* subgroup. The independent predictors of mortality at 14, 30, and 90 days according to multivariate analysis are presented in Table 3.

## 4. Discussion

The findings of the current study highlight key differences in clinical features, risk factors, antifungal resistance, and mortality, contributing to the growing body of literature on candidemia caused by *C. auris* vs. NACS in ICU settings.

Despite *C. auris* being recognized as an emerging multidrug-resistant organism with outbreak potential, our findings demonstrate that 14-, 30-, and 90-day mortality rates were comparable between the *C. auris* and NACS groups. This is in accordance with recent studies suggesting that candidemia outcomes may be more strongly influenced by host-related factors and illness severity than by the specific *Candida* species involved [19,20]. Key host-related determinants associated with adverse outcomes include advanced age; the presence of HM or SOM; CKD; DM; immunosuppressive conditions (including corticosteroid use or neutropenia); the need for MV; CVC; prior broad-spectrum antibiotic or antifungal exposure; and elevated illness severity scores, such as the SOFA score. These factors contribute to an increased vulnerability to candidemia and limit the host’s capacity to mount an effective immune response, which may, in turn, confound the direct contribution to mortality outcomes [19,20]. Previous reports have linked *C. auris* with increased mortality due to delayed diagnosis, limited antifungal options, and underlying comorbidities [21,22]. Nevertheless, large cohort studies, reviews and meta-analyses have challenged the assumption that *C. auris* independently predicts higher mortality, particularly when adequate early treatment is initiated [23,24,25].

In our study, the SOFA score emerged as the most consistent independent predictor of mortality at 14, 30, and 90 days, underscoring its utility in assessing severity in critically ill patients with candidemia. Notably, in the NACS subgroup, the PBS was independently associated with increased 30- and 90-day mortality. Originally developed for multidrug-resistant Gram-negative bacteremia, the PBS has also been validated in candidemia, with both the PBS and SOFA scores shown to predict mortality by reflecting infection severity and underscoring the need for early risk stratification and timely intervention, regardless of *Candida* species [23,26,27]. In *C. auris* cases, univariate analyses identified the SOFA score and the presence of SOM as significant factors for 14-day mortality, and age for 30-day mortality. The significantly older age observed in the NACS group may have influenced clinical outcomes, as older age and SOM have previously been reported as risk factors for mortality in patients with candidemia, suggesting that elderly and immunosuppressed individuals may be particularly vulnerable to poor outcomes regardless of the infecting *Candida* species [28,29]. However, in our multivariate analysis, only the SOFA score remained independently associated with mortality. This discrepancy may suggest that disease severity at presentation is a more robust predictor of mortality than chronological age alone. Alternatively, the non-significance of age may also be related to the limited sample size or differences in host–pathogen interactions that may not be fully captured by conventional severity scoring systems. Although sepsis was more frequently observed at the onset of *C. auris* candidemia, it was independently associated with 30-day mortality only in NACS cases, highlighting that early antifungal treatment and supportive care may mitigate its prognostic impact. Several clinical and laboratory parameters, including but not limited to comorbidity burden, the presence of indwelling catheters, and corticosteroid exposure, did not differ significantly between the *C. auris* and NACS groups in our study. These findings suggest that candidemia severity and risk factors are broadly comparable across species, further supporting the conclusion that host-related factors, rather than species-specific traits, may play a dominant role in determining clinical outcomes.

Fluconazole resistance among *C. auris* isolates exceeded 90% in our study, aligning with national and international surveillance reports and supporting the recommendation of echinocandins as first-line therapy [2,30]. However, unlike some prior studies that reported increased echinocandin and amphotericin B resistance in *C. auris*, our findings do not demonstrate significant differences in resistance rates to these antifungal agents compared to NACS [2,30]. This may reflect regional variations in antifungal susceptibility patterns or differences in local infection control practices that limit the emergence of highly resistant *C. auris* strains. This discrepancy may hypothetically be attributed to regional variations in antifungal susceptibility patterns or the effectiveness of local infection control measures that potentially limit the emergence and spread of highly resistant *C. auris* strains. Similarly, the absence of a significant difference in antifungal adequacy or treatment-related mortality between groups might reflect, albeit speculatively, the timely initiation of appropriate antifungal therapy and the implementation of standardized institutional candidemia management protocols. It is important to note that the uniform empirical use of echinocandins in our ICU settings may have contributed to the overall low impact of antifungal resistance on mortality. However, it is well recognized that delays in antifungal treatment are associated with worse outcomes in candidemia, and continued efforts are needed to enhance early diagnostic strategies and optimize treatment protocols [23,24,25].

Our study revealed a significantly higher rate of prior antifungal exposure—particularly fluconazole—among patients with *C. auris* candidemia compared to those with NACS candidemia patients. Prior antifungal therapy is a well-established contributor to the emergence of *C. auris*, as selective pressure in ICU settings promotes the proliferation of resistant strains [10]. This association likely explains the higher prevalence of fluconazole resistance, prolonged hospitalization, and delayed candidemia onset in the *C. auris* group, findings consistent with previous data [31,32]. The extended ICU stay and increased use of invasive devices, including urinary catheters, further reflect the role of healthcare-associated factors in *C. auris* acquisition.

A growing body of evidence, including our findings, suggests that *C. auris* candidemia is more frequently associated with late recurrence compared to candidemia caused by NACS [33,34]. While early recurrence rates were comparable between both groups in our study, which may suggest similar initial treatment efficacy, the increased risk of late relapse observed in *C. auris* patients could potentially be influenced by factors such as prolonged colonization, biofilm formation, and extended hospital stays, although a direct causal relationship cannot be established based on our data. These findings emphasize the importance of targeted antifungal stewardship to reduce fluconazole overuse, alongside rigorous infection control and potential decolonization strategies to prevent reinfection and limit nosocomial transmission.

## 5. Conclusions

In conclusion, our study highlights key epidemiological and clinical distinctions between *C. auris* and NACS candidemia among critically ill patients. *C. auris* candidemia was associated with significantly higher fluconazole resistance, more frequent prior antifungal exposure, prolonged ICU stay, increased use of urinary catheters, and late recurrence. In contrast, early recurrence, antifungal treatment adequacy, and underlying comorbidities did not differ significantly between the groups. Despite greater healthcare exposure and antifungal resistance in the *C. auris* group, the 14-, 30-, and 90-day mortality rates were comparable between the groups. Multivariate analyses revealed that mortality was primarily driven by illness severity, as measured by the SOFA score, rather than by the infecting *Candida* species. These findings underscore the importance of timely antifungal therapy, robust antimicrobial stewardship, and strict infection control to mitigate recurrence and prevent nosocomial transmission of *C. auris* in ICU settings.

## 6. Limitations

Our study has several limitations. First, its retrospective design may have introduced information bias, particularly in the assessment of clinical variables, such as time to appropriate antifungal therapy initiation. Additionally, due to the lack of post-mortem investigations or standardized adjudication protocols, we were unable to definitively determine whether mortality was directly attributable to candidemia or to underlying comorbidities; therefore, all-cause mortality was assessed in our analysis. Second, the relatively small sample size of *C. auris* cases limits the generalizability of our findings and may have reduced the statistical power to detect certain associations. Third, we did not perform molecular confirmation or genotyping of *C. auris* isolates, which could have provided additional insights into strain-specific resistance patterns and transmission dynamics. Future multicenter studies with larger cohorts and genomic characterization of *C. auris* isolates are warranted to further clarify the clinical impact of this emerging pathogen.

## Figures and Tables

**Table 1:** Demographic and clinical features of all patients and comparison of patients with *C. auris* and NACS candidemia.

	All Patients, n = 182	*Candida auris* Candidemia, n = 33	NACS ^a^ Candidemia, n = 149	*p*-Value
Age, median (Q1–Q3)	69 (59.8–80)	65 (50–71.5)	70 (62–81)	0.003 *
Sex, n (%)				
Male	96 (52.7)	21 (63.6)	75 (50.3)	0.166
Female	86 (47.3)	12 (36.4)	74 (49.7)	
SOFA ^b^ score, median (Q1–Q3)	6 (4–8)	7 (4–8)	6 (4–8)	0.693
PBS ^c^, median (Q1–Q3)	4 (3–6)	5 (3–6)	4 (2–6)	0.827
Comorbidities, n (%)				
DM ^d^	62 (34.1)	12 (36.4)	50 (33.6)	0.758
CVD ^e^	86 (47.3)	15 (45.5)	71 (47.7)	0.819
CKD ^f^	42 (23.1)	4 (12.1)	38 (25.5)	0.099
HM ^g^	8 (4.4)	0 (0)	8 (5.4)	0.372
SOM ^h^	51 (28)	10 (30.3)	41 (27.5)	0.747
Hemodialysis, n (%)	37 (20.3)	7 (21.2)	30 (20.1)	0.889
CRRT ^i^, n (%)	8 (4.4)	2 (6.1)	6 (4)	0.963
History of transplantation, n (%)				
SOT ^j^	8 (4.4)	1 (3)	7 (4.7)	0.490
HSCT ^k^	3 (1.6)	0 (0)	3 (2)	
Indwelling devices, n (%)				
CVC ^l^	167 (91.8)	32 (97)	135 (90.6)	0.393
UC ^m^	148 (81.3)	31 (93.9)	117 (78.5)	0.040 *
MV ^n^	133 (73.1)	24 (72.7)	109 (73.2)	0.960
Abdominal surgery (in previous 30 days), n (%)	16 (8.8)	4 (12.1)	12 (8.1)	0.684
Sepsis, n (%)	89 (48.9)	22 (66.7)	67 (45)	0.024 *
Parenteral nutrition, n (%)	52 (28.6)	7 (21.2)	45 (30.2)	0.301
Corticosteroid treatment (in previous seven days), n (%)	66 (36.3)	10 (30.3)	56 (37.6)	0.431
Non-corticosteroid immunosuppressive treatment (in previous 7 days), n (%)	11 (6)	1 (3)	10 (6.7)	0.690
Antibiotic treatment (in previous 30 days), n (%)	174 (95.6)	33 (100)	141 (94.6)	0.372
Antifungal treatment (in previous 30 days), n (%)				
Fluconazole	32 (17.6)	8 (24.2)	24 (16.1)	0.003 *
Echinocandin	15 (8.2)	8 (24.2)	7 (4.7)	
Amphotericin B	1 (0.5)	0 (0)	1 (0.7)	
Days of hospitalization before candidemia, median (Q1–Q3)	30.5 (15–51.5)	49 (35.5–104)	26 (12–41)	<0.001 *
Days of ICU ^o^ stay before candidemia, median (Q1–Q3)	17 (6–36)	35 (17.5–89.5)	13 (3.5–30)	<0.001 *
Primary infection, n (%) Secondary infection, n (%)	79 (43.4) 103 (56.6)	12 (36.4) 21 (63.6)	67 (45) 82 (55)	0.367
Source of secondary infection, n (%)				
CLABSI ^p^	63 (61.2)	14 (66.7)	49 (59.8)	0.425
IAI ^q^	22 (21.4)	2 (9.5)	20 (24.4)	
CAUTI ^r^	15 (14.6)	4 (19)	11 (13.4)	
SSI ^s^	2 (1.9)	1 (4.8)	1 (1.2)	
SSTI ^t^	1 (1)	0 (0)	1 (1.2)	
Antifungal resistance, n (%)				
Fluconazole	71 (39)	31 (93.9)	40 (26.8)	<0.001 *
Echinocandin	27 (14.8)	3 (9.1)	24 (16.1)	0.450
Amphotericin B	19 (10.4)	5 (15.2)	14 (9.4)	0.507
Time to appropriate antifungal therapy initiation (hours) ^u^, median (Q1–Q3)	48.5 (24–73.5)	48 (24–75)	49 (23–73)	0.925
Time to negative conversion of control blood culture (days) ^v^, median (Q1–Q3)	5 (3.3–8)	5 (4–7)	5 (3–8)	0.720
Recurrence ^w^, n (%)				
Early	11/135	0/26	11/101	0.118
Late	13/53	8/16	5/53	<0.001 *
Mortality, n (%)				
14-day mortality	80 (44)	12 (36.4)	68 (45.6)	0.331
30-day mortality	121 (66.5)	18 (54.5)	103 (69.1)	0.108
90-day mortality	145 (79.7)	24 (72.7)	121 (81.2)	0.273

^a^ NACS: non-*auris Candida* species; ^b^ SOFA: Sequential Organ Failure Assessment; ^c^ PBS: Pitt Bacteremia Score; ^d^ DM: diabetes mellitus; ^e^ CVD: cardiovascular disease; ^f^ CKD: chronic kidney disease; ^g^ HM: hematological malignancy; ^h^ SOM: solid organ malignancy; ^i^ CRRT: continuous renal replacement therapy; ^j^ SOT: solid organ transplantation; ^k^ HSCT: hematopoietic stem cell transplantation; ^l^ CVC: central venous catheter; ^m^ UC: urinary catheter; ^n^ MV: mechanic ventilation; ^o^ ICU: intensive care unit; ^p^ CLABSI: central line-associated bloodstream infection; ^q^ IAI: intra-abdominal infection; *^r^* CAUTI: catheter-associated urinary tract infection; ^s^ SSI: surgical site infection; ^t^ SSTI: skin and soft tissue infection. ^u^ The time to appropriate antifungal therapy initiation (hours) was calculated for all candidemia cases, excluding patients who died before appropriate treatment initiation and those who received appropriate antifungal therapy before a yeast signal was detected in blood culture. The analysis included a total of 130 candidemia cases, comprising 27 cases of *C. auris* candidemia and 103 cases of NACS candidemia. ^v^ The time to negative conversion of control blood culture (days) was calculated for all candidemia cases, excluding patients who did not have a follow-up blood culture and those who died before a negative blood culture result. The analysis was based on 96 candidemia cases, including 21 cases of *C. auris* candidemia and 75 cases of NACS candidemia. ^w^ The recurrence rate was performed based on patients who survived during the relevant time period. * *p* < 0.05.

**Table 2:** Comparison of demographic and clinical characteristics between 30-day survivors and non-survivors, stratified by pathogen group (*C. auris* vs. NACS).

	All Patients			*Candida auris* Candidemia			NACS ^a^ Candidemia		
	Survivors, n = 61	Non-Survivors, n = 121	*p*-Value	Survivors, n = 15	Non-Survivors, n = 18	*p*-Value	Survivors, n = 46	Non-Survivors, n = 103	*p*-Value
Age, median (Q1–Q3)	68 (20.5–74.5)	69 (61.5–82)	0.064	55 (43–65)	67.5 (49.5–72.25)	0.016 *	71 (62–80)	70 (62–84)	0.498
Sex, n (%)									
Male	34 (55.7)	62 (61.2)	0.566	10 (66.7)	11 (61.1)	0.741	24 (52.2)	51 (49.5)	0.764
Female	27 (44.3)	59 (48.8)		5 (33.3)	3 (38.9)		22 (47.8)	52 (50.5)	
SOFA ^b^ score, median (Q1–Q3)	4 (3–6)	6 (4–8.5)	<0.001 *	4 (3–8)	7.5 (5–8.5)	0.062	4 (3–6)	6 (4–9)	0.001 *
PBS ^c^, median (Q1–Q3)	4 (2–5)	5 (3–6)	<0.001 *	4 (3–6)	5 (3–6.5)	0.361	3 (1.75–4)	5 (3–6)	<0.001 *
Comorbidities, n (%)									
DM ^d^	21 (34.4)	41 (33.9)	0.942	4 (26.7)	8 (44.4)	0.287	17 (37)	33 (32)	0.557
CVD ^e^	33 (54.1)	53 (43.8)	0.189	7 (46.7)	8 (44.4)	0.898	26 (56.5)	45 (43.7)	0.147
CKD ^f^	12 (19.7)	30 (24.8)	0.439	1 (6.7)	3 (16.7)	0.369	11 (23.9)	27 (26.2)	0.766
HM ^g^	1 (1.6)	7 (5.8)	0.163	0 (0)	0 (0)	NA	1 (2.2)	7 (6.8)	0.211
SOM ^h^	12 (19.7)	39 (32.2)	0.075	3(20)	7 (38.9)	0.234	9 (19.6)	32 (31.1)	0.146
Hemodialysis, n (%)	11 (18)	26 (21.5)	0.585	3 (20)	4 (22.2)	0.876	8 (17.4)	22 (21.4)	0.577
CRRT ^i^, n (%)	0 (0)	8 (6.6)	0.010 *	0 (0)	2 (11.1)	0.112	0 (0)	6 (5.8)	0.033 *
History of transplantation, n (%)									
SOT ^j^	1 (1.6)	7 (5.8)	0.411	0 (0)	1 (5.6)	0.266	1 (2.2)	6 (5.8)	0.605
HSCT ^k^	1 (1.6)	2 (1.7)		0 (0)	0 (0)		1 (2.2)	2 (1.9)	
Indwelling devices, n (%)									
CVC ^l^	54 (88.5)	113 (93.4)	0.260	14 (93.3)	18 (100)	0.204	40 (87)	95 (92.2)	0.320
UC ^m^	48 (78.7)	100 (82.6)	0.518	13 (86.7)	18 (100)	0.069	35 (76.1)	82 (79.6)	0.628
MV ^n^	43 (70.5)	90 (74.4)	0.577	10 (66.7)	14 (77.8)	0.476	33 (71.7)	76 (73.8)	0.794
Abdominal surgery (in previous 30 days), n (%)	3 (4.9)	13 (10.7)	0.190	1 (6.7)	3 (16.7)	0.369	2 (4.3)	10 (9.7)	0.267
Sepsis, n (%)	26 (42.6)	63 (52.1)	0.229	11 (73.3)	11 (61.1)	0.458	15 (32.6)	52 (50.5)	0.043 *
Parenteral nutrition, n (%)	16 (26.2)	36 (29.8)	0.619	5 (33.3)	2 (11.1)	0.117	11 (23.9)	34 (33)	0.264
Corticosteroid treatment (in previous seven days), n (%)	19 (31.1)	47 (38.8)	0.308	4 (26.7)	6 (33.3)	0.677	15 (32.6)	41 (39.8)	0.402
Non-corticosteroid immunosuppressive treatment (in previous 7 days), n (%)	4 (6.6)	7 (5.8)	0.837	0 (0)	1 (5.6)	0.266	4 (8.7)	6 (5.8)	0.527
Antibiotic treatment (in previous 30 days), n (%)	58 (95.1)	116 (95.9)	0.809	15 (100)	18 (100)	NA	43 (93.5)	98 (95.1)	0.682
Antifungal treatment (in previous 30 days), n (%)									
Fluconazole	11 (18)	21 (17.4)	0.596	3 (20)	5 (27.8)	0.970	8 (17.4)	16 (15.5)	0.917
Echinocandin	5 (8.2)	10 (8.3)		4 (26.7)	4 (22.2)		1 (2.2)	6 (5.8)	
Amphotericin B	1 (1.6)	0 (0)		0 (0)	0 (0)		1 (2.2)	0 (0)	
Days of hospitalization before candidemia, median (Q1–Q3)	35 (14.5–65)	30 (15–49)	0.420	71 (40–121)	45.5 (32–81.25)	0.274	24.5 (10.5–49.5)	28 (13–40)	0.721
Days of ICU ^o^ stay before candidemia, median (Q1–Q3)	20 (5.5–41)	15 (6–34)	0.205	41 (18–104)	34 (15.75–56.75)	0.381	14 (2.75–34.5)	13 (5–28)	0.666
Primary infection, n (%)	25 (41)	54 (44.6)	0.640	4 (26.7)	8 (44.4)	0.290	21 (45.7)	46 (44.7)	0.910
Secondary infection, n (%)	36 (59)	67 (55.4)		11 (73.3)	10 (55.6)		25 (54.3)	57 (55.3)	
Source of secondary infection, n (%)									
CLABSI ^p^	23 (63.9)	40 (59.7)	0.643	9 (81.8)	5 (50)	0.378	14 (56)	35 (61.4)	0.937
IAI ^q^	7 (19.4)	15 (22.4)		0 (0)	2 (20)		7 (28)	13 (22.8)	
CAUTI ^r^	5 (13.9)	10 (14.9)		1 (9.1)	3 (30)		4 (16)	7 (12.3)	
SSI ^s^	1 (2.8)	1 (1.5)		1 (9.1)	0 (0)		0 (0)	1 (1.8)	
SSTI ^t^	0 (0)	1 (1.5)		0 (0)	0 (0)		0 (0)	1 (1.8)	
Time to appropriate antifungal therapy initiation (hours) ^u^, median (Q1–Q3)	48.5 (26.25–72)	48.5 (22.25–75.75)	0.985	48 (24–75)	49.5 (24.5–79.75)	0.981	49 (28–72)	48.5 (22–75.75)	0.932
Time to negative conversion of control blood culture (days) ^v^, median (Q1–Q3)	6 (4–9)	5 (3–6)	0.046 *	5 (4–8.75)	5 (4–6)	0.754	6 (4–9.5)	5 (3–6.25)	0.057

^a^ NACS: non-*auris Candida* species; ^b^ SOFA: Sequential Organ Failure Assessment; ^c^ PBS: Pitt Bacteremia Score; ^d^ DM: diabetes mellitus; ^e^ CVD: cardiovascular disease; ^f^ CKD: chronic kidney disease; ^g^ HM: hematological malignancy; ^h^ SOM: solid organ malignancy; ^i^ CRRT: continuous renal replacement therapy; ^j^ SOT: solid organ transplantation; ^k^ HSCT: hematopoietic stem cell transplantation; ^l^ CVC: central venous catheter; ^m^ UC: urinary catheter; ^n^ MV: mechanic ventilation; ^o^ ICU: intensive care unit; ^p^ CLABSI: central line-associated bloodstream infection; ^q^ IAI: intra-abdominal infection; *^r^* CAUTI: catheter-associated urinary tract infection; ^s^ SSI: surgical site infection; ^t^ SSTI: skin and soft tissue infection. ^u^ The time to appropriate antifungal therapy initiation (hours) was calculated for all candidemia cases, excluding patients who died before appropriate treatment initiation and those who received appropriate antifungal therapy before a yeast signal was detected in blood culture. The analysis included a total of 130 candidemia cases, comprising 27 cases of *C. auris* candidemia and 103 cases of NACS candidemia. ^v^ The time to negative conversion of control blood culture (days) was calculated for all candidemia cases, excluding patients who did not have a follow-up blood culture and those who died before a negative blood culture result. The analysis was based on 96 candidemia cases, including 21 cases of *C. auris* candidemia and 75 cases of NACS candidemia. * *p* < 0.05.

**Table 3:** Independent predictors of mortality at 14, 30, and 90 days in multivariate analysis.

Mortality	Groups	Variable	Adjusted OR ^a^ (95%CI)	*p*-Value
14-day mortality	All patients	SOFA ^c^	1.305 (1.153–1.477)	<0.001 *
		PBS ^d^	1.069 (0.936–1.220)	0.324
		Sepsis	1.384 (0.680–2.818)	0.370
	*C. auris* candidemia	SOFA	1.378 (1.210–1.558)	<0.001 *
		SOM ^e^	2.034 (0.868–4.767)	0.102
		Days of ICU stay before candidemia	0.999 (0.985–1.013)	0.883
	NACS ^b^ candidemia	SOFA	1.254 (1.097–1.433)	0.001 *
		PBS	1.093 (0.945–1.264)	0.233
		Sepsis	2.339 (1.069–5.118)	0.033 *
30-day mortality	All patients	SOFA	1.138 (1.016–1.273)	0.025 *
		PBS	1.163 (1.015–1.334)	0.030 *
		CRRT ^f^	0.986 (0.050–19.455)	0.993
	*C. auris* candidemia	Age	1.057 (1.000–1.116)	0.050
	NACS candidemia	SOFA	1.108 (0.968–1.268)	0.136
		PBS	1.202 (1.027–1.407)	0.022 *
		Sepsis	1.238 (0.535–2.867)	0.618
		CRRT	1.463 (0.234–9.150)	0.684
90-day mortality	All patients	Sex	1.683 (0.771–3.672)	0.191
		SOFA	1.121 (0.978–1.284)	0.102
		PBS	1.258 (1.064–1.488)	0.007 *
	NACS candidemia	SOFA	1.150 (0.967–1.367)	0.113
		PBS	1.270 (1.047–1.541)	0.015 *
		Sepsis	1.198 (0.432–3.318)	0.729
		CRRT	1.276 (0.185–11.224)	0.772

^a^ OR: odds ratio; ^b^ NACS: non-*auris Candida* species; ^c^ SOFA: Sequential Organ Failure Assessment; ^d^ PBS: Pitt Bacteremia Score; ^e^ SOM: solid organ malignancy; ^f^ CRRT: continuous renal replacement therapy. * *p* < 0.05.

## Data Availability

The data that support the findings of this study are available upon request from the corresponding author. The data are not publicly available due to privacy or ethical restrictions.

## Supplementary Materials

The following supporting information can be downloaded at: https://www.mdpi.com/article/10.3390/jof11080552/s1, Table S1: Comparison of demographic and clinical characteristics between 14-day survivors and non-survivors, stratified by pathogen group (*C. auris* vs. NACS); Table S2: Comparison of demographic and clinical characteristics between 90-day survivors and non-survivors, stratified by pathogen group (*C. auris* vs. NACS).
