# Supplementary material for: Candida auris vs. Non-Candida auris Candidemia in Critically Ill Patients: Clinical Outcomes, Risk Factors, and Mortality
Source: J Fungi (Basel). 2025 Jul 24;11(8):552. doi: 10.3390/jof11080552 (PMC12387453; doi:10.3390/jof11080552)
Supplement: Supplementary file 1 [file jof-11-00552-s001.zip › jof-3693409-supplementary.pdf]

Journal of *Fungi*

MDPI

Table S1. Comparison of demographic and clinical characteristics between 14-day survivors and non-survivors, stratified by pathogen group (*C. auris* vs. NACS).

	All patients			*Candida auris* candidemia			NACS[a] candidemia		
	**Survivors, n=102**	**Non-survivors, n=80**	**p-value**	**Survivors, n=21**	**Non-survivors, n=12**	**p-value**	**Survivors, n=81**	**Non-survivors, n=68**	**p-value**
Age, median (Q1-Q3)	69 (54.75-80)	68.5 (61-78)	0.551	58 (45-70.5)	67 (61.5-72.75)	0.096	71 (62.5-81.5)	69 (61-80.5)	0.816
Sex, n (%)									
Male	54 (52.9)	42 (52.5)	0.953	13 (38.1)	8 (66.7)	0.784	41 (50.6)	34 (50)	0.940
Female	48 (47.1)	38 (47.5)		8 (61.9)	4 (33.3)		40 (49.4)	34 (50)	
SOFA[b] score, median (Q1-Q3)	4 (3-6)	8 (5.25-10)	<0.001*	4 (3-8)	8 (6.25-10)	0.011*	4 (3-6)	7.5 (5-10)	<0.001*
PBS[c], median (Q1-Q3)	4 (2-6)	5.5 (3-6.75)	0.001*	4 (3-6)	5 (3-7.5)	0.726	4 (2-6)	6 (3-6.75)	<0.001*
Comorbidities, n (%)									
DM[d]	37 (36.3)	25 (31.3)	0.478	6 (28.6)	6 (50)	0.221	31 (38.3)	19 (27.9)	0.183
CVD[e]	53 (52)	33 (41.3)	0.151	9 (42.9)	6 (50)	0.692	44 (54.3)	27 (39.7)	0.075
CKD[f]	20 (19.6)	22 (27.5)	0.210	2 (9.5)	2 (16.7)	0.552	18 (22.2)	20 (29.4)	0.316
HM[g]	2 (2)	6 (7.5)	0.070	0 (0)	0 (0)	NA	2 (2.5)	6 (8.8)	0.083
SOM[h]	23 (22.5)	28 (35)	0.063	3 (14.3)	7 (58.3)	0.008*	20 (24.7)	21 (30.9)	0.399
Hemodialysis, n (%)	19 (18.6)	18 (22.5)	0.519	6 (28.6)	1 (8.3)	0.148	13 (16)	17 (25)	0.175
CRRT[i], n (%)	3 (2.9)	5 (6.3)	0.281	1 (4.8)	1 (8.3)	0.685	2 (2.5)	4 (5.9)	0.290
History of transplantation, n (%)									
SOT[j]	2 (2)	6 (7.5)	0.076	0 (0)	1 (8.3)	0.149	2 (2.5)	5 (7.4)	0.147
HSCT[k]	1 (1)	2 (2.5)		0 (0)	0 (0)		1 (1.2)	2 (2.9)	
Indwelling devices, n (%)									
CVC[l]	93 (91.2)	74 (92.5)	0.747	20 (95.2)	12 (100)	0.337	73 (90.1)	62 (91.2)	0.826
UC[m]	82 (80.4)	66 (82.5)	0.717	19 (90.5)	12 (100)	0.170	63 (77.8)	54 (79.4)	0.809
MV[n]	72 (70.6)	61 (76.3)	0.393	15 (71.4)	9 (75)	0.824	57 (70.4)	52 (76.5)	0.403
Abdominal surgery (in previous 30 days), n (%)	7 (6.9)	9 (11.3)	0.300	1 (4.8)	3 (25)	0.092	6 (7.4)	6 (8.8)	0.752
Sepsis, n (%)	38 (37.3)	51 (63.7)	<0.001*	15 (71.4)	7 (58.3)	0.446	23 (28.4)	44 (64.7)	<0.001*
Parenteral nutrition, n (%)	28 (27.5)	24 (30)	0.706	6 (28.6)	1 (8.3)	0.148	22 (27.2)	23 (33.8)	0.378
Corticosteroid treatment (in previous seven days), n (%)	34 (33.3)	32 (40)	0.353	6 (28.6)	4 (33.3)	0.775	28 (34.6)	28 (41.2)	0.407
Non-corticosteroid immunosuppressive treatment (in previous 7 days), n (%)	4 (3.9)	7 (8.8)	0.176	0 (0)	1 (8.3)	0.149	4 (4.9)	6 (8.8)	0.346

Antibiotic treatment (in previous 30 days), n (%)	98 (96.1)	76 (95)	0.726	21 (100)	12 (100)	NA	77 (95.1)	64 (94.1)	0.799
Antifungal treatment (in previous 30 days), n (%)<br>Fluconazole<br>Echinocandin<br>Amphotericin B	19 (18.6)<br>9 (8.8)<br>1 (1)	13 (16.3)<br>6 (7.5)<br>0 (0)	0.416	4 (19)<br>5 (23.8)<br>0 (0)	4 (33.3)<br>3 (25)<br>0 (0)	0.583	15 (18.5)<br>4 (4.9)<br>1 (1.2)	9 (13.2)<br>3 (4.4)<br>0 (0)	0.292
Days of hospitalization before candidemia, median (Q1-Q3)	31.5 (14.75-64.25)	29.5 (15.25-41)	0.382	71 (41-121)	42.5 (28-52)	0.069	25 (11-51)	28 (12.25-40)	0.850
Days of ICU[o] stay before candidemia, median (Q1-Q3)	18.5 (6-40)	16 (6-33.75)	0.416	41 (25-108.5)	26 (8.25-44.5)	0.044*	13 (4-29)	14.5 (3-31.75)	0.804
Primary infection, n (%)<br>Secondary infection, n (%)	41 (40.2)<br>61 (59.8)	38 (47.5)<br>42 (52.5)	0.324	7 (33.3)<br>14 (66.7)	5 (41.7)<br>7 (58.3)	0.633	34 (42)<br>47 (58)	33 (48.5)<br>35 (51.5)	0.940
Source of secondary infection, n (%)<br>CLABSI[p]<br>IAI[q]<br>CAUTI[r]<br>SSI[s]<br>SSTI[t]	37 (60.7)<br>12 (19.7)<br>9 (14.8)<br>2 (3.3)<br>1 (1.6)	26 (61.9)<br>10 (23.8)<br>6 (14.3)<br>0 (0)<br>0 (0)	0.650	10 (71.4)<br>0 (0)<br>3 (21.4)<br>1 (7.1)<br>0 (0)	4 (57.1)<br>2 (28.6)<br>1 (14.3)<br>0 (0)<br>0 (0)	0.480	27 (57.4)<br>12 (25.5)<br>6 (12.8)<br>1 (2.1)<br>1 (2.1)	22 (62.9)<br>8 (22.9)<br>5 (14.3)<br>0 (0)<br>0 (0)	0.383
Time to appropriate antifungal therapy initiation (hours)[u], median (Q1-Q3)	50.5 (28-75)	45 (18.25-71.5)	0.097	54 (25.5-85.5)	28.5 (16.75-50.5)	0.085	49 (31-72.5)	47 (16.75-75.25)	0.327
Time to negative conversion of control blood culture (days)[v], median (Q1-Q3)	5 (4-9)	5 (3-6)	0.067	5 (4.5-8.5)	4.5 (2.25-6)	0.317	5.5 (3-9)	5 (3-6)	0.172

[a]NACS: non-*auris Candida* species; [b]SOFA: Sequential Organ Failure Assessment; [c]PBS: Pitt Bacteremia Score; [d]DM: diabetes mellitus; [e]CVD: cardiovascular disease; [f]CKD: chronic kidney disease; [g]HM: hematological malignancy; [h]SOM: solid organ malignancy; [i]CRRT: continuous renal replacement therapy**;** [j]SOT: solid organ transplantation**;** [k]HSCT: hematopoietic stem cell transplantation; [l]CVC: central venous catheter; [m]UC: urinary catheter; [n]MV: mechanic ventilation; [o]ICU: intensive care unit; [p]CLABSI*:* central line-associated bloodstream infection; [q]IAI*:* intra-abdominal infection; [r]CAUTI: catheter-associated urinary tract infection; [s]SSI: surgical site infection; [t]SSTI: skin and soft tissue infection. [u]The time to appropriate antifungal therapy initiation (hours) was calculated for all candidemia cases, excluding patients who died before appropriate treatment initiation and those who received appropriate antifungal therapy before a yeast signal was detected in blood culture. The analysis included a total of 130 candidemia cases, comprising 27 cases of *C. auris* candidemia and 103 cases of NACS candidemia. [v]The time to negative conversion of control blood culture (days) was calculated for all candidemia cases, excluding patients who did not have a follow-up blood culture and those who died before a negative blood culture result. The analysis was based on 96

candidemia cases, including 21 cases of *C. auris* candidemia and 75 cases of NACS candidemia. *p<0.05.

Table S2. Comparison of demographic and clinical characteristics between 90-day survivors and non-survivors, stratified by pathogen group (*C. auris* vs. NACS).

	**All patients**			***Candida auris* candidemia**			**NACS[a] candidemia**		
	**Survivors, n=37**	**Non-survivors, n=145**	**p-value**	**Survivors, n=9**	**Non-survivors, n=24**	**p-value**	**Survivors, n=28**	**Non-survivors, n=121**	**p-value**
Age, median (Q1-Q3)	65 (50.5-73.5)	70 (61-81)	0.031*	55 (46-63)	67 (52.75-72.75)	0.059	69 (54.5-79.75)	71 (63-83.5)	0.210
Sex, n (%)									
Male	22 (59.5)	74 (51)	0.360	6 (66.7)	15 (62.5)	0.824	16 (57.1)	59 (48.8)	0.424
Female	15 (40.5)	71 (49)		3 (33.3)	9 (37.5)		12 (42.9)	62 (51.2)	
SOFA[b] score, median (Q1-Q3)	4 (3-6)	6 (4-8)	0.002*	5 (3.5-9)	7 (4-8)	0.890	4 (2.25-5)	6 (4-8.5)	0.001*
PBS[c], median (Q1-Q3)	3 (1-4)	5 (3-6)	<0.001*	4 (3-5)	5 (3-6)	0.290	3 (1-4)	5 (3-6)	<0.001*
Comorbidities, n (%)									
DM[d]	11 (29.7)	51 (35.2)	0.533	2 (22.2)	10 (41.7)	0.289	9 (32.1)	41 (33.9)	0.860
CVD[e]	20 (54.1)	66 (45.5)	0.353	4 (44.4)	11 (45.8)	0.943	16 (57.1)	55 (45.5)	0.264
CKD[f]	7 (18.9)	35 (24.1)	0.501	0 (0)	4 (16.7)	0.097	7 (25)	31 (25.6)	0.946
HM[g]	1 (2.7)	7 (4.8)	0.910	0 (0)	0 (0)	NA	1 (3.6)	7 (5.8)	0.623
SOM[h]	5 (13.5)	46 (31.7)	0.028*	1 (11.1)	9 (37.5)	0.297	4 (14.3)	37 (30.6)	0.067
Hemodialysis, n (%)	5 (13.5)	32 (22.1)	0.248	1 (11.1)	6 (25)	0.361	4 (14.3)	26 (21.5)	0.392
CRRT[i], n (%)	0 (0)	8 (4.4)	0.312	0 (0)	2 (8.3)	0.941	0 (0)	6 (5)	0.110
History of transplantation, n (%)									
SOT[j]	0 (0)	8 (5.5)	0.138	0 (0)	1 (4.2)	0.421	0 (0)	7 (5.8)	0.789
HSCT[k]	1 (2.7)	2 (1.4)		0 (0)	0 (0)		1 (3.6)	2 (1.7)	
Indwelling devices, n (%)									
CVC[l]	31 (83.8)	136 (93.8)	0.067	8 (88.9)	24 (100)	0.101	23 (82.1)	112 (92.6)	0.114
UC[m]	28 (75.7)	120 (82.8)	0.324	8 (88.9)	23 (95.8)	0.481	20 (71.4)	97 (80.2)	0.310
MV[n]	23 (62.2)	110 (75.9)	0.094	7 (77.8)	17 (70.8)	0.686	16 (57.1)	93 (76.9)	0.034*
Abdominal surgery (in previous 30 days), n (%)	3 (8.1)	13 (9)	0.868	1 (11.1)	3 (12.5)	0.913	2 (7.1)	10 (8.3)	0.842
Sepsis, n (%)	14 (37.8)	75 (51.7)	0.131	6 (66.7)	16 (66.7)	1	8 (28.6)	59 (48.8)	0.053
Parenteral nutrition, n (%)	7 (18.9)	45 (31)	0.145	3 (33.3)	4 (16.7)	0.312	4 (14.3)	41 (33.9)	0.042*
Corticosteroid treatment (in previous seven days), n (%)	11 (29.7)	55 (37.9)	0.354	4 (44.4)	6 (25)	0.288	7 (25)	49 (40.5)	0.127
Non-corticosteroid immunosuppressive treatment (in previous 7 days), n (%)	2 (5.4)	9 (6.2)	0.853	0 (0)	1 (4.2)	0.421	2 (7.1)	8 (6.6)	0.920
Antibiotic treatment (in previous 30 days), n (%)	35 (94.6)	139 (95.9)	1	9 (100)	24 (100)	NA	26 (92.9)	115 (95)	0.656

Antifungal treatment (in previous 30 days), n (%)									
Fluconazole	7 (18.9)	25 (17.2)		2 (22.2)	6 (25)		5 (17.9)	19 (15.7)	
Echinocandin	4 (10.8)	11 (7.6)	0.282	3 (33.3)	5 (20.8)	0.498	1 (3.6)	6 (5)	0.406
Amphotericin B	1 (2.7)	0 (0)		0 (0)	0 (0)		1 (3.6)	0 (0)	
Days of hospitalization before candidemia, median (Q1-Q3)	31 (13-60.5)	30 (15-51)	0.832	71 (37.5-124)	45.5 (34.5-101.5)	0.370	24.5 (9.5-39.75)	28 (12.5-43)	0.447
Days of ICU[o] stay before candidemia, median (Q1-Q3)	15 (4-38.5)	17 (6-35.5)	0.958	41 (31.5-108.5)	34 (15.5-81)	0.328	11 (1.25-27.75)	15 (5.5-32.5)	0.309
Primary infection, n (%)	16 (43.2)	63 (43.4)	0.982	3 (33.3)	9 (37.5)	0.824	13 (46.4)	54 (44.6)	0.863
Secondary infection, n (%)	21 (56.8)	82 (56.6)		6 (66.7)	15 (62.5)		15 (53.6)	67 (55.4)	
Source of secondary infection, n (%)									
CLABSI[p]	12 (57.1)	51 (62.2)		5 (83.3)	9 (60)		7 (46.7)	42 (62.7)	
IAI[q]	6 (28.6)	12 (14.6)		0 (0)	2 (13.3)		6 (40)	14 (20.9)	
CAUTI[r]	3 (14.3)	16 (19.5)	0.729	1 (16.7)	3 (20)	0.497	2 (13.3)	9 (13.4)	0.314
SSI[s]	0 (0)	2 (2.4)		0 (0)	1 (6.7)		0 (0)	1 (1.5)	
SSTI[t]	0 (0)	1 (1.2)		0 (0)	0 (0)		0 (0)	1 (1.5)	
Time to appropriate antifungal therapy initiation (hours)[u], median (Q1-Q3)	48 (28-72.25)	49 (22-75)	0.651	36 (25.5-73.5)	52.5 (24-84.75)	0.900	50 (31-72.5)	48.5 (21.75-75)	0.528
Time to negative conversion of control blood culture (days)[v], median (Q1-Q3)	5 (3.75-8.25)	5 (3-8)	0.621	5 (4.75-6.5)	5 (4-9)	1	5.5 (3-9.75)	5 (3-8)	0.554

[a]NACS: non-*auris Candida* species; [b]SOFA: Sequential Organ Failure Assessment; [c]PBS: Pitt Bacteremia Score; [d]DM: diabetes mellitus; [e]CVD: cardiovascular disease; [f]CKD: chronic kidney disease; [g]HM: hematological malignancy; [h]SOM: solid organ malignancy; [i]CRRT: continuous renal replacement therapy; [j]SOT: solid organ transplantation; [k]HSCT: hematopoietic stem cell transplantation; [l]CVC: central venous catheter; [m]UC: urinary catheter; [n]MV: mechanic ventilation; [o]ICU: intensive care unit; [p]CLABSI: central line-associated bloodstream infection; [q]IAI: intra-abdominal infection; [r]CAUTI: catheter-associated urinary tract infection; [s]SSI: surgical site infection; [t]SSTI: skin and soft tissue infection. [u]The time to appropriate antifungal therapy initiation (hours) was calculated for all candidemia cases, excluding patients who died before appropriate treatment initiation and those who received appropriate antifungal therapy before a yeast signal was detected in blood culture. The analysis included a total of 130 candidemia cases, comprising 27 cases of *C. auris* candidemia and 103 cases of NACS candidemia. [v]The time to negative conversion of control blood culture (days) was calculated for all candidemia cases, excluding patients who did not have a follow-up blood culture and those who died before a negative blood culture result. The analysis was based on 96 candidemia cases, including 21 cases of *C. auris* candidemia and 75 cases of NACS candidemia. *$p<0.05$.
